# Insight into the Viscoelasticity of Self-Assembling Smectic Liquid Crystals of Colloidal Rods from Active Microrheology Simulations

**DOI:** 10.1021/acs.jctc.3c00356

**Published:** 2023-06-30

**Authors:** Fabián A. García Daza, Antonio M. Puertas, Alejandro Cuetos, Alessandro Patti

**Affiliations:** † Department of Physical, Chemical and Natural Systems, Pablo de Olavide University, 41013 Sevilla, Spain; ‡ Department of Chemical Engineering, The University of Manchester, Manchester M13 9PL, United Kingdom; § Department of Chemistry and Physics, University of Almeriá, 04120 Almería, Spain; ¶ Department of Applied Physics, University of Granada, Avenida Fuente Nueva s/n, 18071 Granada, Spain

## Abstract

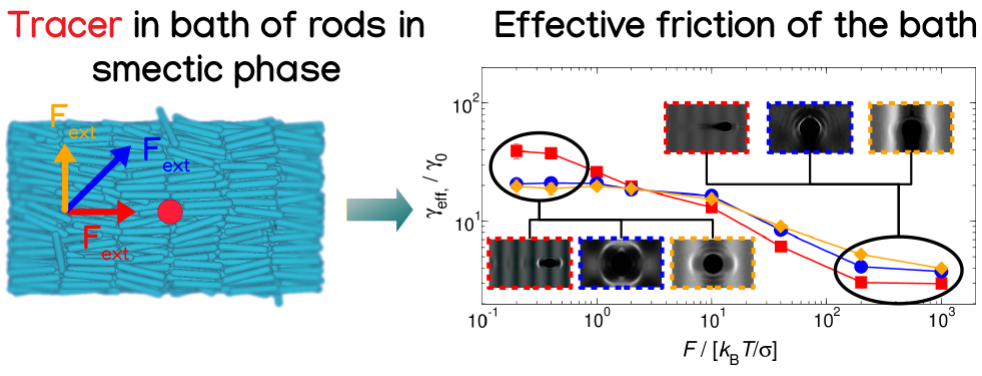

The rheology of colloidal suspensions is of utmost importance in a wide variety of interdisciplinary applications in formulation technology, determining equally interesting questions in fundamental science. This is especially intriguing when colloids exhibit a degree of long-range positional or orientational ordering, as in liquid crystals (LCs) of elongated particles. Along with standard methods, microrheology (MR) has emerged in recent years as a tool to assess the mechanical properties of materials at the microscopic level. In particular, by active MR one can infer the viscoelastic response of a soft material from the dynamics of a tracer particle being dragged through it by external forces. Although considerable efforts have been made to study the diffusion of guest particles in LCs, little is known about the combined effect of tracer size and directionality of the dragging force on the system’s viscoelastic response. By dynamic Monte Carlo simulations, we apply active MR to investigate the viscoelasticity of self-assembling smectic (Sm) LCs consisting of rodlike particles. In particular, we track the motion of a spherical tracer whose size is varied within a range of values matching the system’s characteristic length scales and being dragged by constant forces that are parallel, perpendicular, or at 45° to the nematic director. Our results reveal a uniform value of the effective friction coefficient as probed by the tracer at small and large forces, whereas a nonlinear, force-thinning regime is observed at intermediate forces. However, at relatively weak forces the effective friction is strongly determined by correlations between the tracer size and the structure of the host fluid. Moreover, we also show that external forces forming an angle with the nematic director provide additional details that cannot be simply inferred from the mere analysis of parallel and perpendicular forces. Our results highlight the fundamental interplay between tracer size and force direction in assessing the MR of Sm LC fluids.

## 1 Introduction

Colloidal dispersions are a class of soft materials whose structural and mechanical characteristics make them particularly suitable for a wide range of applications, including nanomedicine,^1^ biology,^2^ robotics,^3^ and the food industry,^4^ among others. In the case of fluids consisting of nonspherical particles, at sufficiently large density, their constituent particles can orient and form long-ranged ordered mesophases, referred to as liquid crystals (LCs), whose structural properties strongly depend on particle anisotropy. Most commonly, ordering in LCs is described by the nematic director, **n**, which formally gathers the average orientation of the particles. Orientational ordering can be accompanied by positional ordering as in smectic (Sm) LCs, consisting of stacks of liquidlike layers of oriented prolate particles.^5^ Smectics are characterized not only by a very peculiar morphology but also by a very interesting dynamics with the particles rattling and jumping following quasiquantized layer-to-layer hops.^6−8^ Due to their intrinsic anisotropy, Sm fluids are expected to exhibit a space-dependent rheology, with the viscosity in the direction parallel to the nematic director different from that in the directions perpendicular to it. Such directional viscoelastic behaviors offer a rich spectrum of mechanical properties of technological and scientific interest. It is therefore important to understand the rheology of these systems from a fundamental perspective. A variety of techniques can be implemented to obtain the elastic and viscous properties of LCs,^9^ including macroscopic (or bulk) rheology as well as active and passive microrheology (MR).^10−13^

The aim of MR is to derive the viscoelastic properties of the host medium on the basis of the dynamics of one or more guest tracer (or probe) particles embedded into it. In passive MR,^14−16^ the friction and the viscoelastic moduli of the medium can be derived by monitoring the free diffusion of the tracer, whose motion is controlled by thermal fluctuations of the particles in the host phase. Thereby, it provides access to the linear viscoelastic response of the bath. By contrast, in active MR^12,17−20^ an external stimulus on the tracer forces it to displace throughout the bath and probe its linear and nonlinear viscoelastic responses. Particularly, in constant-force active MR^17,21^ the tracer is pulled through the host phase by a constant force, **F**, and develops a long-time steady velocity ⟨**v**_*t*_⟩ which is related to the effective friction coefficient of the bath, γ_eff_, through the Stokes’ drag law **F** = γ_eff_⟨**v**_*t*_⟩. Within this context, theoretical^17−19,22^ and simulation^21,23−26^ works have reported the occurrence of two plateaus for the effective friction at low and high forces and a force-thinning regime representing the nonlinear response of the bath where the friction decreases with the external force. Interestingly enough, despite the significant number of works on the diffusion of guest particles in colloidal dispersions,^27−37^ only a limited number of them^38−45^ focus on extracting the viscoelastic properties of LC phases from the dynamic response of the tracer particles. Nonetheless, from an active MR standpoint, little is known on how these properties in Sm LCs may vary with the size of the tracer when this is of the order of the characteristic length scales of the bath particles^46^ or what occurs to the mechanical response of the Sm phases when the external force is not completely parallel or perpendicular to the nematic director.

In this work, we tackle these open questions by simulating the dynamics of a spherical tracer pulled by an external constant force across a bath of colloidal rods in the Sm phase. Our specific goal is to determine the effect of orientational and positional order on the effective friction coefficient of colloidal Sm LCs. In particular, we are interested in how the directionality of the external force exerted on the tracer particle influences the microrheology of Sm LCs. More specifically, we study three distinct scenarios in which the external force is aligned in parallel, perpendicular, and diagonal directions relative to the preferred orientation of the particles, marked by the nematic director **n**. Furthermore, we also investigate the impact of the tracer size relative to the characteristic length scale of the Sm phase on the linear and nonlinear viscoelastic responses of the bath.

To this end, we employ the dynamic Monte Carlo (DMC) technique which has been developed to study the Brownian motion of colloidal particles under the most general conditions.^47−52^ More recently, the DMC has been extended to investigate the passive^45^ and fixed-force active^25,26^ MR of colloidal suspensions. Formally, the DMC is a Metropolis-based technique that, in the limit of small displacements and rotations, mimics the Brownian motion of particles and reproduces time-dependent events. Its main advantage over other methods like Brownian dynamics (BD) or molecular dynamics (MD) simulations consists of being able to reproduce the thermal motion of particles without solving deterministic or stochastic equations of motion. Consequently, the DMC allows exploration of both short- and long-time-scale dynamics of particles, the latter being particularly useful for systems exhibiting long-range structural relaxation times, as in the case of LCs.

Our paper is organized as follows: in the next section, a brief description of the DMC method for the study of fixed-force active MR is given as well as the main characteristics of Sm colloidal LCs and tracer particles studied in this work. In Section 3, we discuss the effect of the tracer size and the direction of the external force with respect to the nematic director on the viscoelastic properties of smectic LC phases. We report for the different tracers the linear and nonlinear responses of the system’s effective friction coefficient for forces parallel, transverse, and diagonal to **n**. Finally, in Section 4, we summarize our most important findings.

## 2 Model and Methods

**Figure 1:**
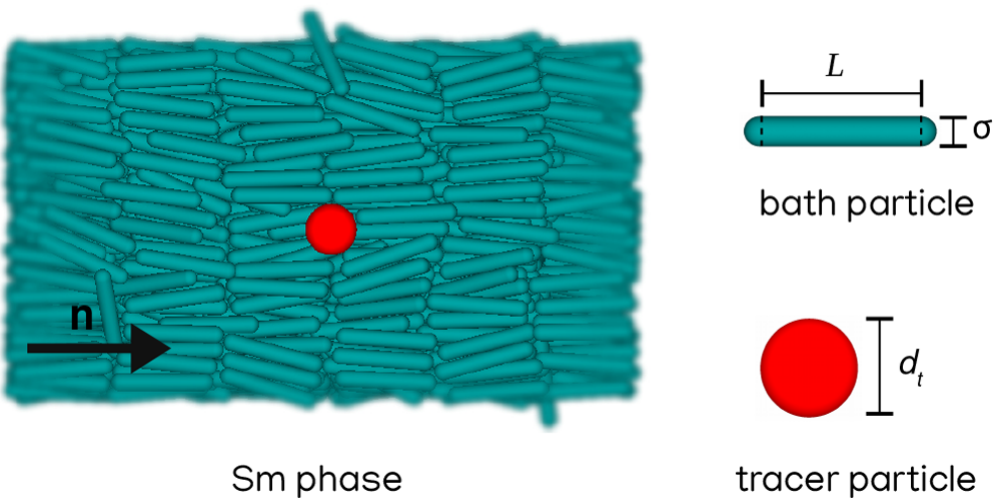
Side view of 1400 spherocylinders of length-to-diameter ratio *L*/σ + 1 = 6 and one spherical tracer of diameter *d*_*t*_ = 3σ in a Sm phase. The black arrow indicates the orientation of the nematic director **n**.

In this work, we have studied the active MR of a colloidal suspension of *N*_*r*_ = 1400 rods, modeled as spherocylinders, in Sm LCs and *N*_*t*_ = 1 tracer particle. While bath particles have a length-to-diameter ratio L* ≡ *L*/σ + 1 = 6, the tracer is modeled as a sphere with diameter 1σ ≤ *d*_*t*_ ≤ 3σ. A typical representation of the system under study is shown in Figure 1. In our simulations interparticle interactions are modeled by a hard-core potential. Length, energy, and time are given in units of σ, *k*_B_*T*, and τ = σ^2^/*D*_0_, respectively, with *k*_B_ Boltzmann’s constant, *T* the absolute temperature, *D*_0_ = *k*_B_*T*/(η_*s*_σ) a diffusion constant, and η_*s*_ the viscosity of the solvent (not modeled explicitly). In this work, we are particularly interested in the dynamical response of the tracer particle when an external constant force **F** pulls it through the host fluid. Before running DMC simulations to study the system’s dynamics, we perform a series of Monte Carlo (MC) simulations in the canonical ensemble to equilibrate the systems at a volume fraction of ϕ = 0.51 that corresponds to a stable Sm phase.^53^ The volume fraction is defined by ϕ = (*N*_*r*_*v*_*r*_ + *N*_*t*_*v*_*tr*_)/*V*, where *v*_*r*_ = πσ^3^/6 + πσ^2^L*/4, *v*_*tr*_ = π*d*_*t*_^3^/6, and *V* refer, respectively, to the rod, tracer, and simulation box volumes. Since interparticle interactions are mediated via a hard-core potential, attempted moves are always accepted unless an overlap occurs. To detect the occurrence of an overlap between the rods, we employed the algorithm proposed by Vega and Lago.^54^ Equilibration was achieved when the nematic (*S*_2_) and smectic (λ) order parameters reached steady values within statistical fluctuations. The nematic order parameter is obtained from the diagonalization of the following symmetric tensor:
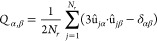
1where α, β refer to the spatial coordinates {*x*, *y*, *z*}, **û**_*jα*_ are the unit vectors that indicate the orientation of particle *j*, and δ_αβ_ is the Kronecker delta. The largest eigenvalue resulting from the diagonalization of *Q*_α,β_ provides the order parameter *S*_2_, while the corresponding eigenvector gives the nematic director **n**. The smectic order parameter reads
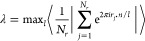
2where *l* is the value of the layer spacing that maximizes λ, **r**_*j*_ is the position vector of particle *j*, and *i* is the imaginary unit. In our equilibrium MC simulations, we obtained *S*_2_ ≈ 0.9 and λ ≈ 0.8, thereby confirming the formation of the Sm phase. We also note that the presence of the tracer particle does not have a tangible effect on the values of the order parameters, regardless of its size. In addition, simulations were conducted on systems with varying numbers of particles to examine the occurrence of finite size effects. However, it was found that these effects were not present within the range of tracer radii studied, likely due to their suppression by the long-range structure. This is confirmed by our analysis of density maps of the rods, as depicted in Figure 3, where it is appreciated that the size of the trailing depleted zone behind the tracer remains within the borders of the simulation box, irrespective of the applied external forces on the probe particle. With this, we can conclude that our simulation box size exceeded the persistence length (*l*_p_). Subsequently, the equilibrium configurations obtained were used to perform production DMC simulations under the canonical ensemble.

The DMC method mimics the Brownian motion of the system’s particles by displacing selected random particles according to the standard Metropolis algorithm. However, to obtain more realistic trajectories, unphysical moves like jumps, swaps, or cluster moves are not allowed. In what follows, we review the aspects of DMC that are relevant to the present study and refer the reader to refs (25 and 47−50) for additional details. We also stress that the DMC technique in its current form does not incorporate an explicit solvent, and hence fluid-mediated hydrodynamic interactions (HI) between particles are not considered. While HI are generally assumed to be negligible compared to the interparticle interactions engendered by the almost ceaseless, direct collisions between particles in especially dense systems, we are also aware that HI effects can be relevant in active MR of colloidal crystals.^55^ In a DMC cycle, we perform *N* = *N*_*r*_ + *N*_*t*_ independent random attempts to displace and rotate the particles, rotations being exclusive for the rods. The moves are accepted or rejected according to a Metropolis algorithm with probability min[1, e^–βΔ*E*^], where β ≡ (*k*_B_*T*)^−1^ and Δ*E* is the change in energy due to the movement of the particle. The position of a rod particle is updated by decoupling its displacement into three contributions δ**r**_*r*_ = *X*_∥_**û**_*r*_ + *X*_⊥,1_**v̂**_*r*,1_ + *X*_⊥,2_**v̂**_*r*,2_, **û**_*r*_ being a unit vector parallel to the main rod axis, whereas **v̂**_*r*,*m*_ with *m* = 1, 2, represent two random unitary vectors perpendicular to **û**_*r*_ and to each other. The magnitude of a rod displacement is selected at random from uniform distributions that satisfy |*X*_∥_| ≤ δ*r*_∥_ and |*X*_⊥,*m*_| ≤ δ*r*_⊥_. The maximum elementary displacements δ*r*_∥_ and δ*r*_⊥_ are linked to the translational diffusivities at infinite dilution through the following equations:
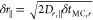
3
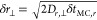
4where *D*_*r*,∥_, *D*_*r*,⊥_ and *t*_MC,*r*_ represent, respectively, the parallel and perpendicular diffusion coefficient of a rod particle at infinite dilution and its time step in the MC time scale. For rotations, the orientation vector varies as δ**û**_*r*_ = *Y*_φ,1_**ŵ**_*r*,1_ + *Y*_φ,2_**ŵ**_*r*,2_, where the vectors **ŵ**_*r*,*m*_ are randomly chosen in such a way that they are perpendicular to each other and to **û**_*r*_. The maximum rotations are chosen to satisfy |*Y*_φ,*m*_| ≤ *δφ*, where
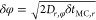
5with *D*_r,φ_ being the rotational diffusion coefficient of the rod at infinite dilution. In the case of the spherical tracer, rotations are ignored, and only translational moves are considered. The displacement of the tracer reads δ**r**_*t*_ = *X*_∥_^*t*^**û**_*t*_ + *X*_⊥,1_^*t*^**v̂**_*t*,1_ + *X*_⊥,2_^*t*^**v̂**_*t*,2_, where **û**_*t*_ is a unit vector parallel to the external force and **v̂**_*t*,*m*_ are two random vectors perpendicular to each other and to **û**_*t*_. The displacements of the tracer fulfill the conditions |*X*_∥_^*t*^| ≤ δr_∥_^*t*^ and |*X*_⊥,*m*_^*t*^| ≤ δr_⊥_^*t*^. While the displacements along the external force must include an extra term, its parallel counterpart is ruled exclusively by Brownian forces. Specifically
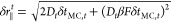
6
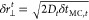
7where *F* = |**F**| and *D*_*t*_ and δ*t*_MC,*t*_ are the tracer diffusion coefficient at infinite dilution and its MC time step, respectively.

The translational and rotational diffusion coefficients of rodlike particles have been calculated from the analytical expressions derived from the induced-force method proposed by Bonet Avalos and co-workers:^56^
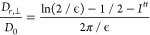
8
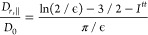
9
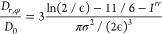
10where ϵ^–1^ = 2(L* + 1), , and , with *h*(*x*) = (1 – 2*x*^2*n*^)^1/2*n*^ a parametric function that models particles with symmetry of revolution. The characteristic shape of the spherocylinders is well reproduced by taking *n* = 8. Consequently *I*^*tt*^ ≃ −0.0061, and *I*^*rr*^ ≃ −0.017. The specific values of the translational and rotational diffusion coefficients at infinite dilution are, respectively, *D*_*r*,⊥_ = 3.560 × 10^–2^*D*_0_, *D*_*r*,∥_ = 4.467 × 10^–2^*D*_0_, and *D*_*r*,φ_ = 6.020 × 10^–3^ σ^–2^*D*_0_. On the other hand, the diffusion coefficient of the spherical tracer particle with diameter *d*_*t*_ ≥ 1σ is estimated from the Stokes–Einstein equation and reads
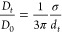
11The maximum displacements and rotations of the particles are set by rods and tracer time steps, δ*t*_MC,*r*_ and δ*t*_MC,*t*_, respectively. Following our recent work,^25^ the time steps of the bath and tracer particles are related through their acceptance rates by the following relationship:
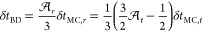
12where δ*t*_BD_ is the elementary time step in the BD scale, while  and  are, respectively, the acceptance rates of bath and tracer particles. Essentially, eq 12 states that while tracers and rods possess different time scales, a rescaling by their corresponding acceptance rates results in the same BD time scale. We stress, however, that for eq 12 to be valid the condition β*F*δ*r*_∥_^*t*,max^ ≪ 1 must be fulfilled, where δ*r*_∥_^*t*,max^ refers to the maximum displacement of the tracer particle along the direction of the external force.^25^ This indicates, according to eq 6, that while for small forces the MC time step of the tracer can fall within a wide range of values, for large forces the value of the time step must decrease. One can then fix the MC time step of either host or tracer particles and determine the other via eq 12 by performing a preliminary trial-and-error DMC simulation. In this work, we have kept constant the time step of the bath, δ*t*_MC,*r*_, in the range 10^–7^τ–10^–3^τ depending on the intensity of the force. Subsequently, the time step of the tracer, δ*t*_MC,*t*_, was calculated by updating every 500 MC cycles the corresponding acceptance rates in eq 12. It should be noted that this preliminary procedure is essential to define a unique BD time scale, which in turns permits to study the systems’ dynamical properties consistently on this time scale.

In this work, we are interested in active MR under fixed-force conditions of a bath of rods in the Sm phase as perceived by a spherical tracer with diameter 1σ ≤ *d*_*t*_ ≤ 3σ. To this end, we evaluate the Stokes’ drag relationship:
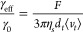
13where γ_0_ = 3*πη*_*s*_*d*_*t*_ is the friction coefficient of the medium, while ⟨*v*_*t*_⟩ = |⟨**v**_*t*_⟩| is the tracer mean velocity parallel to the external force at long times. Following equilibration, during the DMC production stage the tracer is subjected to an external force with a fixed orientation in relation to the nematic director **n**. In this study, we have considered the cases where the force is parallel (**F**_∥_: **n**·**F** = *F*), perpendicular (**F**_⊥_: **n**·**F** = 0), and oriented 45° (**F**_45°_: **n**·**F** = *F* cos(45°)) with respect to **n**. In all cases, we have calculated the long-time mean velocity of the tracer, ⟨*v*_*t*_⟩, from the slope of the averaged tracer displacements over BD time, *t*_BD_. Between 2 × 10^5^ and 6 × 10^6^ MC cycles per trajectory were performed in our simulations depending on the direction of the force. All properties were averaged over 500 uncorrelated time trajectories for each direction of the external force and size of the tracer particle.

## 3 Results and Discussion

In this section, we study the effect of increasing the tracer size on the linear and nonlinear viscoelastic responses of a structured fluid via active MR. To this end, we have simulated a colloidal dispersion of rodlike particles, modeled here as hard spherocylinders, forming a Sm LC phase. To explore the active MR of the host phase, we embedded a spherical tracer particle with diameter *d*_*t*_ within the system. More specifically, while we have chosen a fixed length-to-diameter ratio of L* = 5 for the bath particles, the diameter of the tracer is allowed to vary from 1σ to 3σ. At this particular value of L*, hard rods form stable Sm LCs^53^ for the selected volume fraction ϕ = 0.51. Details of the systems studied in this section are provided in Table S1 of the Supporting Information. To study the effect of the external force on the effective friction coefficient, we have considered forces parallel, **F**_∥_, perpendicular, **F**_⊥_, and diagonal, **F**_45°_, to the nematic director and their magnitude to vary between 0.2*k*_B_*Tσ*^–1^ and 10^3^*k*_B_*Tσ*^–1^.

### 3.1 Effect of External Forces Parallel to the Nematic Director

**Figure 2:**
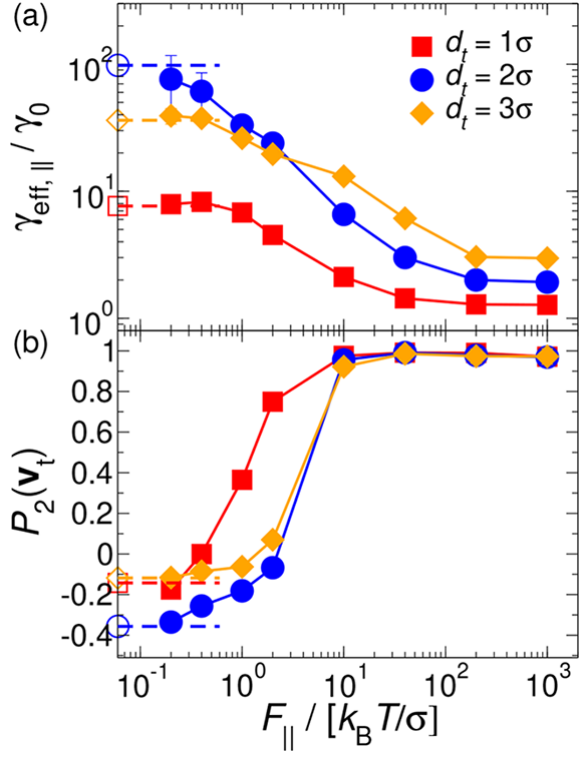
Frame (a): effective friction coefficient of hard rods in Sm phases as a function of the external force pulling the probe particle in a direction parallel to the nematic director, for different tracer diameters, as shown in the legend. Frame (b): averaged correlation function at long times between the unit vectors of the tracer’s velocity and the external force parallel to **n**. While solid symbols and lines represent DMC simulation results, empty symbols and dashed lines refer to results obtained with passive MR (*F* = 0) from ref (45). Solid and dashed lines are guides for the eye.

The influence of an external force **F**_∥_ pointing in the direction of the nematic director **n** on the effective friction is depicted in frame (a) of Figure 2 for different sizes of the tracer. In this figure, the term γ_eff,∥_ is the effective friction coefficient of the system in the direction parallel to **n**. For small forces (*F*_∥_ ≤ 0.4*k*_B_*Tσ*^–1^), the appearance of a plateau is observed for tracers of sizes *d*_*t*_ = 1σ and 3σ. In this regime, the diffusion of the tracer has no tangible effect on the bath–particle equilibrium configurations. Consequently, there is a linear relationship between the external force being applied to the tracer and its steady-state velocity, yielding a constant value of γ_eff,∥_. This low-force linear regime approaches the case of passive MR where external forces are absent and thermal motion drives the particle dynamics.^17^ For comparison, we have included the results of the inverse diffusion coefficient, obtained from DMC simulations as reported in our previous work^45^ for the case *F* = 0, which corresponds to the case of passive MR. In general, we observe an excellent quantitative agreement of the effective friction coefficient estimated from active (solid symbols) and passive (empty symbols) MR. By contrast, for the tracer with diameter *d*_*t*_ = 2σ, where the friction coefficient is largest, a linear regime was not reached at low forces. In this range of forces, our simulations show that the tracer dynamics is strongly restricted by its preference to reside in the interstitial spaces between the Sm layers. As such, weak external forces cannot drive the probe particle through the layers and develop a long-time steady velocity that allows it to reach a linear response relative to γ_eff,∥_.

As the force increases, advection of bath particles gradually prevails over their thermal motion. The force pulling the tracer is sufficiently intense to cause a microstructural deformation of the particle distribution around the probe, developing *force thinning*, where the effective friction decreases with *F*_∥_. This force-thinning regime has been observed in experiments^57,58^ and simulations^12,18,21,23,25,26^ and predicted by theory,^17−19,22,59^ and it resembles shear thinning in viscoelastic fluids, where the shear viscosity decreases as the shear rate increases. Interestingly, while γ_eff,∥_^2σ^ > γ_eff,∥_^3σ^ at small forces, we notice that γ_eff,∥_^2σ^ < γ_eff,∥_^3σ^ for *F*_∥_ ≥ 2*k*_B_*Tσ*^–1^. We believe that this change in trend may be caused by the quantitative differences in the distribution of bath particles that are in contact with the probes. According to Squires and Brady,^17^ the changes in effective friction probed by the tracer follow from Δγ_eff_ ∼ (*Pe*)^−1^∫*n*_*x*_*g*(1;*d*_*t*_)dΩ, where *n*_*x*_ represents the direction of the external force, *g*(1;*d*_*t*_) is the tracer-bath particle pair distribution function at contact, and dΩ is the solid angle of integration. Accordingly, since the number of layered rods in contact with the tracer increases with its size, it is expected to find an increased number of collisions for the largest probe and thus a higher friction perceived by it. To support this hypothesis, we calculated the averaged local concentration of bath particles (ρ(1;*d*_*t*_) ∝ *g*(1;*d*_*t*_)) at contact. While at *F*_∥_ = 0.2*k*_B_*Tσ*^–1^ our simulations revealed that ρ(1;2σ) ≈ 1.11σ^–3^ and ρ(1;3σ) ≈ 0.84σ^–3^, when *F*_∥_ = 10^3^*k*_B_*Tσ*^–1^, the density at the front of the tracer is approximately 0.013σ^–3^ for ρ(1;2σ) and 0.022σ^–3^ for ρ(1;3σ). This suggests that at weak forces it is easier for the bath particles to come into contact with a tracer of diameter *d*_*t*_ = 2σ than with one of *d*_*t*_ = 3σ, and the effective friction probed by the tracers will be larger for the tracer of size *d*_*t*_ = 2σ. By contrast, at large forces a tracer particle of size 3σ is more likely to collide with a rod compared to a tracer with *d*_*t*_ = 2σ. Consequently, the effective friction probed by the tracers will be larger for larger particles. Finally, at high-force intensities, Brownian motion can no longer counterbalance the advective flow of bath particles, and a second Newtonian plateau is observed.

To gain insight into the response of the tracer to the external force, we calculated the correlation function *P*_2_(**v**_*t*_) between the unit orientation vectors of the tracer velocity (**v̂**_*t*_) at long times and the external force (**F̂**) exerted on it
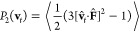
14If the tracer displacement is perpendicular to the external force, it is expected that *P*_2_(**v**_*t*_) ≈ – 1/2. On the other hand, it is expected that *P*_2_(**v**_*t*_) ≈ 1 when the effect of the force on the probe is sufficiently strong to provoke a motion of the tracer in the direction parallel to **F**. Frame (b) of Figure 2 depicts the correlation function *P*_2_(**v**_*t*_) when the force **F**_∥_ is applied on tracers of different sizes. At low forces *P*_2_(**v**_*t*_) approaches −1/2 for the tracer of size *d*_*t*_ = 2σ as a result of its preference to occupy the volumes in the Sm interlayers, which favors its diffusive motion in planes perpendicular to both **n** and **F**_∥_. By contrast, for sizes *d*_*t*_ = 1σ and 3σ there is an increased probability of probing the layers and hence the tracers to respond to the effect of the external force. As a result, a slight rise in *P*_2_(**v**_*t*_) is anticipated, surpassing the −1/2 value and indicating a diffusion across the layers. Subsequently, as the magnitude of **F**_∥_ increases, the tracer moves predominantly in the same direction as the external force, leading to a progressive increase in *P*_2_(**v**_*t*_) until a steady value, *P*_2_(**v**_*t*_) ≃ 1, is reached. It should be noted, however, that for the tracer with diameter *d*_*t*_ = 1σ, *P*_2_(**v**_*t*_) exceeds the values found for *d*_*t*_ = 2σ and 3σ at intermediate forces. In contrast to the larger tracers, the smallest tracer penetrates the different layers most easily through the diffusion channels due to a combined effect of its size and the external force acting on it. This not only causes its velocity **v**_*t*_ to align more effortlessly with **F**_∥_ resulting in an increase in *P*_2_(**v**_*t*_) but also leads to a lower perceived opposition of the surrounding host fluid to its motion as corroborated by its lower effective friction coefficients in frame (a) of Figure 2.

**Figure 3:**
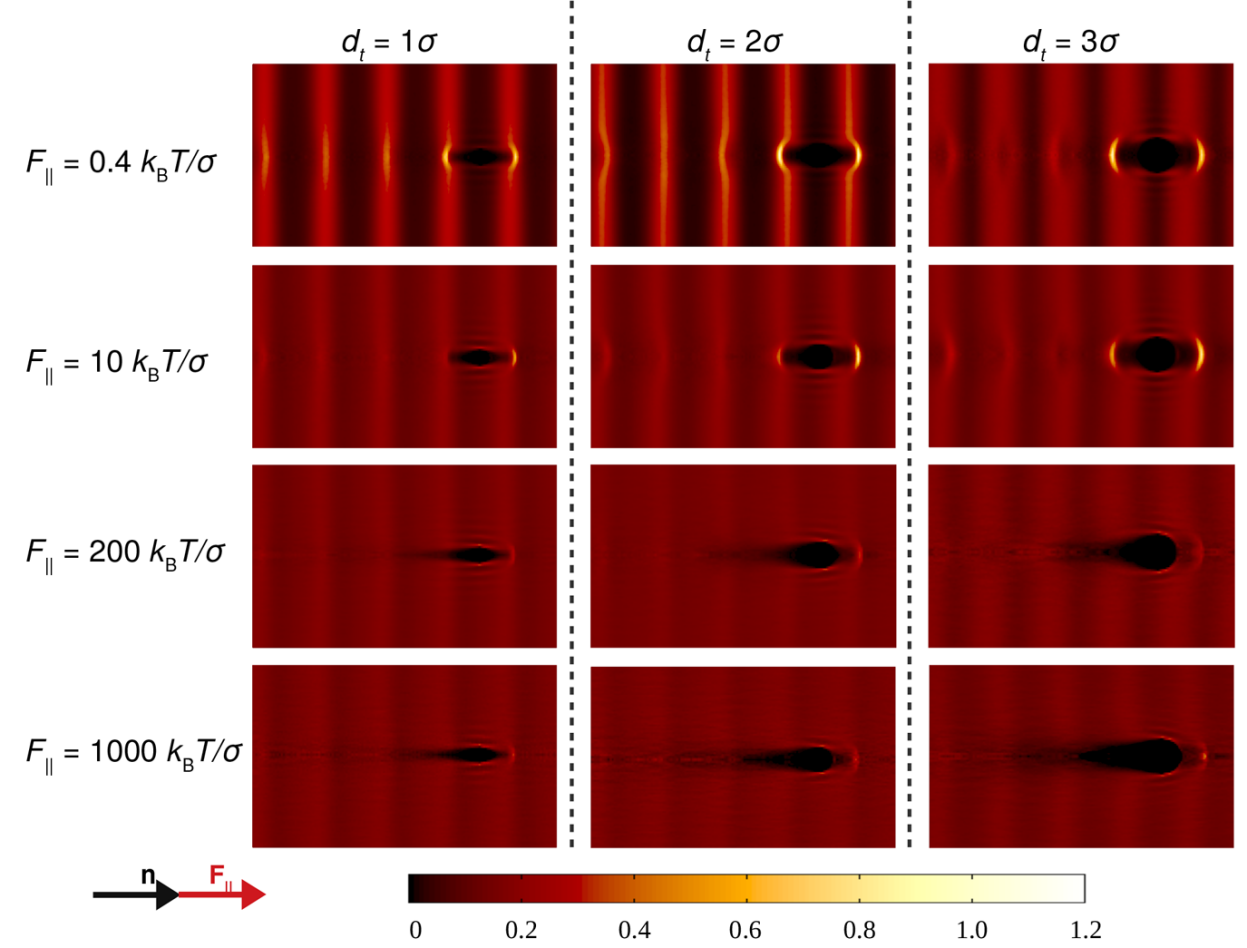
Density maps of a system of hard rods in the Sm phase at ϕ = 0.51 around a tracer with diameter 1σ (left column), 2σ (center column), and 3σ (right column). The tracer particle is pulled by an external force **F**_∥_ parallel to the nematic director **n**. The color palette is shown at the bottom of the figure.

In Figure 3, we present the local densities of bath particles around the tracer. Here, the density is defined as ρ(*v*) = ⟨*N*_*r*_(*v*, *t*)/*v*⟩, where *v* is the local volume defined by virtual rings centered along the tracer axis and parallel to the external force, *N*_*r*_(*v*, *t*) is the number of rods in volume *v* at time *t*, and ⟨···⟩ indicates time average. At small forces, Brownian motion is prevalent, and the symmetry of the bath particle distribution in the vicinity of the tracer remains undisturbed. This scenario replicates that observed in systems in the absence of external forces (passive MR). In this regime the distribution of rods is segmented into light areas representing the different Sm layers perceived by the tracer. While the layered regions become broader when switching from tracers of diameters 1σ to 3σ due to their ability to permeate and diffuse through them,^31,45^ for *d*_*t*_ = 2σ these zones become narrower due to the preference of the tracer to reside in between the Sm layers.

As the force increases, advection becomes comparable to diffusion, and force thinning is induced. In this regime we observe a breaking of the symmetry of the bath particle distribution around the tracer. Moreover, at long forces a soft layer of rods forms in front of the tracer whereas a trailing wake free of particles develops back behind it. In essence, the advection of bath particles dominates the microstructural deformation, and Brownian motion no longer easily dissipates flow energy to heal structural distortions. Additionally, the low-density wake increases gradually with the force for all tracer sizes. This characteristic behavior is similar to that reported in experiments,^57,60^ theoretical studies,^17,22^ and simulations of hard spheres,^21^ rods,^25,38^ and cuboidal^26^ particles. We also noticed that as the force grows, the tracer gradually fails to perceive the order of the microstructured fluid as the layered and interlayered regions become less distinguishable (bottom rows in Figure 3). Analogous results were found in passive MR^45^ of liquid-crystalline phases of rodlike particles. There, the analysis of the systems’ global elastic (*G′*) and viscous (*G″*) moduli and loss ratio (*R* = *G″*/*G′*) revealed a tendency for convergence as the diameter of the tracer increases. This suggested a reduced ability of the tracer to perceive the order of the surrounding medium by increasing its size.

We also have monitored the changes in the orientation of the bath particles in the vicinity of the tracer. Our calculations do not show a strong coordinated ordering of rods surrounding the tracer as shown in Figure S1 of the Supporting Information. Most probably, this limited rotation of host particles is a consequence of the packing of the Sm phase. Since the rods are similarly oriented and distributed in layers, the energy transmitted by the tracer to the surrounding rods is easily dissipated throughout the in-layer particles. Consequently, any attempt to rotate the rods around the tracer will be rapidly counteracted by their close neighbors, causing the particles to reorient only modestly. If the system were less dense, beyond a specific tracer size and force direction, the rods could reorient and even form clusters as reported by Wensink and Löwen^38^ in constant-velocity BD simulations of rodlike particles in nematic LC phases.

### 3.2 Effect of Forces Not Aligned with the Nematic Director

**Figure 4:**
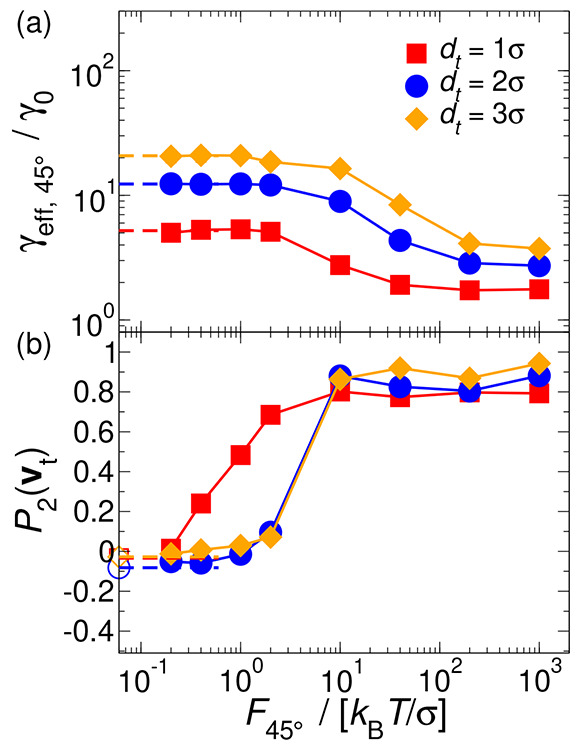
Frame (a): effective friction coefficient of hard rods in the Sm phase as a function of the tracer diameter and the external force pulling the probe oriented 45° to the nematic director. Frame (b): averaged correlation function at long times between the unit vectors of the tracer’s velocity and the external force *F*_45°_. Solid symbols and lines represent DMC simulation results. Dashed lines indicate the location of the plateau at low forces. Solid and dashed lines are guides for the eye.

In the previous section, we have studied the effect of external forces exerted on the tracer pointing in the direction of **n**. Now we turn our attention to the case when the forces are no longer parallel to **n**. Frame (a) of Figure 4 depicts the effective friction γ_eff,45°_ as probed by the tracer when the external force is oriented 45° with respect to the nematic director. In particular, small- and large-force Newtonian plateaus and a force-thinning regime at intermediate forces are found. This behavior is comparable to that observed in frame (a) of Figure 2 for all tracer sizes except for the *d*_*t*_ = 2σ case where a linear regime is not achieved at low *F*_∥_. However, there are quantitative differences with respect to the friction coefficients γ_eff,∥_^1σ^ and γ_eff,∥_^3σ^ presented in frame (a) of Figure 2. Notably, for the case *F*_45°_ the first linear regime persists up to *F*_45°_ ≈ 2*k*_B_*Tσ*^–1^, whereas in the case of forces parallel to **n** this regime extends up to *F*_∥_ ≈ 0.4*k*_B_*Tσ*^–1^. This suggests that for *F*_45°_ when the tracer permeates its closest layers, its motion is more hampered than in the *F*_∥_ case since the number of nearest layered rods that collide with it increases considerably. By contrast, in the parallel case the appearance of diffusion channels along **n** reduces the number of rods in contact with the tracer and eases its transport in this direction.^31^ Accordingly, one can expect that for *F*_45°_ it is more difficult for the tracer to provoke microstructural deformations than to allow it to leave the linear regime while developing force thinning. Figure S2 of the Supporting Information depicts the density profiles of bath particles around the tracer for different intensities of *F*_45°_. While at low forces there is a symmetric distribution of rods around the probe, we note a break in the symmetry of the bath density near the tracer due to force thinning, with an emergence of a subtle low-density wake behind the tracer which becomes more pronounced with its size. Yet, we did not observe any significant change in the orientations of the rods located in the vicinity of the tracer.

Frame (b) of Figure 4 illustrates the correlation function, *P*_2_(**v**_*t*_), between the tracer’s velocity and the external force *F*_45°_. At the lowest force, *P*_2_(**v**_*t*_) ≃ 0 due to the dominance of the tracer’s thermal motion over the external forcing. Eventually, as the force increases, the tracer with *d*_*t*_ = 1σ responds more effectively to *F*_45°_ by aligning its motion to the external force, which also results in lower effective friction, as shown in frame (b) of Figure 4. It is interesting to note that, similarly to the case of the parallel force, at intermediate and large values of *F*_45°_ there is a marked increase in *P*_2_(**v**_*t*_). Nevertheless, the correlation function does not reach unity since the motion of the probe diagonal to **n** prevents it from moving through the in-layer diffusion channels, contrary to the case of *F*_∥_. It is therefore expected that the rods in the Sm layers exert a stronger opposition to the tracer motion such that *P*_2_(**v**_*t*_) < 1 in this force regime.

**Figure 5:**
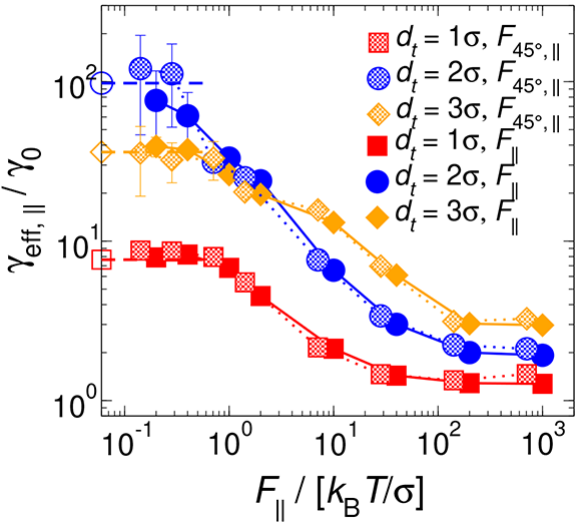
Effective friction coefficient of hard rods in the Sm phase as a function of the tracer diameter and the external force on the probe in the direction parallel to the nematic director **n**. Solid symbols and lines indicate results from DMC simulations when the force is completely parallel to **n**, whereas dotted symbols and lines mark the contribution of the parallel (*F*_45°,∥_) components to **n** of the force oriented 45° to **n**. Empty symbols and dashed lines refer to results obtained with passive MR (*F* = 0) from ref (45). Solid, dashed, and dotted lines are guides for the eye.

In light of these considerations, we now discuss the behavior of the effective friction coefficient parallel and perpendicular to **n** as obtained from the analysis of *F*_45°_. To this end, we have analyzed separately the directional velocity of the tracer (⟨*v*_*t*_⟩_45°,*M*_) in its components parallel (*M* = ∥) and perpendicular (*M* = ⊥) to the nematic director. Within this framework, the directional effective friction γ_eff,*M*_ can be obtained by relating the decomposed external force *F*_45°,*M*_ to its corresponding velocity ⟨*v*_*t*_⟩_45°,*M*_ via eq 13. Figure 5 shows the effective friction along **n** obtained from *F*_45°,∥_ (dotted symbols). For comparison, we have also included the results when a force *F*_∥_ is being applied exclusively along **n** (solid symbols). In both cases, an excellent quantitative agreement is observed within the error bars for all tracer sizes in the force-changing γ_eff,∥_ in both linear and nonlinear regimes. The former is close at small *F*_45°,∥_ to the results in the absence of external forces of passive MR^45^ (empty symbols). Interestingly, contrary to the case of *F*_∥_, from the analysis of *F*_45°,∥_ for the tracer with size *d*_*t*_ = 2σ, the Newtonian linear regime at low forces is captured and compares to the observations from passive MR. An external force diagonal to **n** triggers a local misalignment of neighboring bath particles, which allows the tracer to overcome the energy barrier to penetrate the Sm layers. This permits, particularly at low forces, the tracer to explore the bath–particle configurations more easily and assess their viscoelastic properties.

**Figure 6:**
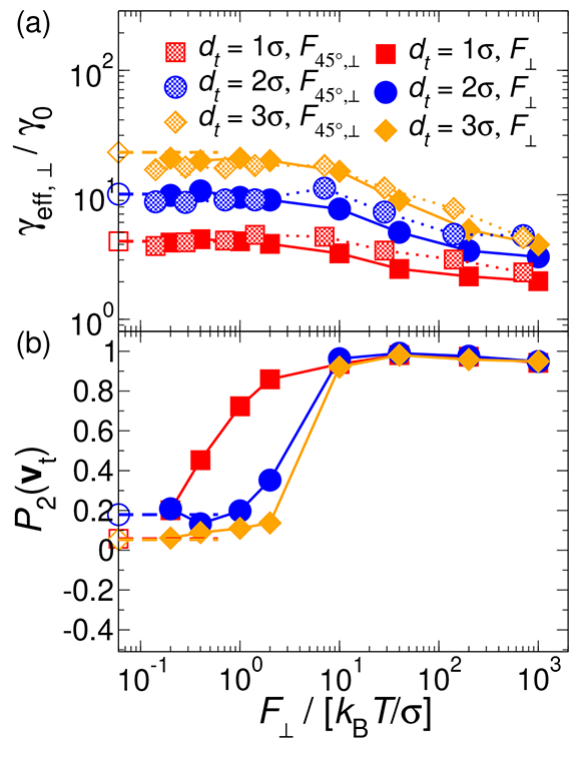
Frame (a): effective friction coefficient of hard rods in the Sm phase as a function of the tracer diameter and the external force on the probe in the direction perpendicular to the nematic director **n**. Frame (b): averaged correlation function at long times between the unit vectors of the tracer’s velocity and the external force *F*_⊥_. Solid symbols and lines refer to the results from DMC simulations when the force is entirely perpendicular to **n**, whereas dotted symbols and lines mark the contribution of the perpendicular *F*_45°,⊥_ components to **n** of the force oriented 45° to **n**. Empty symbols and dashed lines refer to results obtained with passive MR (*F* = 0) from ref (45). Solid, dashed, and dotted lines are guides for the eye.

Equally interesting is the response of the effective friction in the direction perpendicular to **n**. Frame (a) of Figure 6 presents γ_eff,⊥_ as probed by the tracer when a force *F*_⊥_ perpendicular to **n** is exerted on it (solid symbols). While a Newtonian plateau is reached at small and large forces, a force thinning at intermediate forces is observed with γ_eff,⊥_^3σ^ > γ_eff,⊥_^2σ^ > γ_eff,⊥_^1σ^. The same qualitative behavior as that reported for γ_eff,∥_ and γ_eff,45°_ in frames (a) of Figures 2 and 4, respectively, is observed. It should be noted, however, that this similarity is not valid in the case of *d*_*t*_ = 2σ for *F*_∥_ as the tracer faces difficulty in exhibiting a linear response at low force intensities. At low *F*_⊥_, the intensity of Brownian motion prevents structural distortions in the arrangement of bath particles around the tracer. This results in a linear response of the bath to the external stimulus yielding a constant value of γ_eff,⊥_ that approaches the passive MR limit (empty symbols). As the force increases, it is noted that the force thinning arises from a value *F*_⊥_ > 2*k*_B_*Tσ*^–1^ that exceeds that found when the external force is parallel to **n** (*F*_∥_ > 0.4*k*_B_*Tσ*^–1^). This suggests that it is more difficult for the tracer to deform the surrounding microstructure when an external force perpendicular to **n** acts on it. Consequently, the advective coupling of the bath particles to the motion of the tracer is hindered which affects the extent of the linear regime. Finally, for large values of the external force, γ_eff,⊥_ displays a second plateau, where advection dominates the microstructural deformation of the embedding medium. Furthermore, from the bath–particle density profiles in Figure S3 of the Supporting Information we note a symmetrical distribution of rods around the tracer at low forces, which is subsequently interrupted as the force increases, ending up with the appearance of a trailing depletion region behind the tracer for large forces.

The correlation functions, *P*_2_(**v**_*t*_), between the tracer velocities and *F*_⊥_ for the three case studies of probe sizes, are presented in frame (b) of Figure 6. For small forces, *P*_2_(**v**_*t*_) is higher for *d*_*t*_ = 2σ, evidencing its preference to move parallel to *F*_⊥_ within the interlayered volumes. However, as the force gradually increases, the tracer with size *d*_*t*_ = 1σ exhibits a higher *P*_2_(**v**_*t*_) due to its ability to move perpendicular to **n** either within the Sm layers or in the quasi-2D interlayer volumes. It is worth noting that, similar to the case of *F*_45°_ in frame (b) of Figure 4, the convergence of *P*_2_(**v**_*t*_) ≃ 1 occurs with the appearance of the nonlinear regime in γ_eff,⊥_ at *F*_⊥_ ≥ 10 (see frame (a) of Figure 6). This suggests a close coupling between the direction of the tracer particle velocity and *F*_⊥_ at intermediate and large force intensities, favored to some extent by the inhomogeneities of the Sm phase.

We also computed the effective viscosity γ_eff,⊥_ from relating *F*_45°,⊥_ to the tracer velocity ⟨*v*_*t*_⟩_45°,⊥_ transverse to the nematic director. The resulting values of γ_eff,⊥_ are shown in frame (a) of Figure 6 (dotted symbols). At low-force conditions the estimates from *F*_45°,⊥_ are quantitatively similar to those arising from applying a net force *F*_⊥_ on the particle. Interestingly, once it reaches the nonlinear regime, γ_eff,⊥_ calculated from *F*_45°,⊥_ exceeds the reference values obtained by *F*_⊥_. As already reported in the literature, in the absence of external forces, the tracer prefers to occupy the quasi-2D spacing between contiguous layers^48,61,62^ with occasional displacements from one interlayer to the next through fast hops.^31,45^ Moreover, from our DMC simulations we observe that this characteristic behavior is preserved at small forces that are within the linear viscoelastic regime. Hence, as the force *F*_45°_ increases beyond the linear regime, it drives the tracer to visit and diffuse across the Sm layers much more regularly compared to *F*_⊥_. Consequently, it can be expected that the tracer will probe a larger γ_eff,⊥_ when calculated from *F*_45°,⊥_ than *F*_⊥_. We note, however, that these differences are reduced for the tracer of size *d*_*t*_ = 3σ. In the zero-force limit, the tracer undergoes a smoother and more progressive diffusion along the different layers as reported in the past.^45^ This helps the tracer to probe more homogeneously the mechanical response of the microstructured medium. Therefore, the impact of exerting *F*_45°_ or *F*_⊥_ on the tracer will have a marginal effect on γ_eff,⊥_^3σ^ as confirmed in frame (a) of Figure 6. It should be emphasized that the results presented so far suggest that, following the application of a force diagonal to **n**, the tracer surpasses more easily the energy barrier imposed by the correlations between its size and the Sm layer arrangements that prevent it from mapping the bath–particle configurations. Once this barrier is overcome, the tracer permeates the Sm layers more frequently to probe more effectively the viscoelastic properties of the fluid. Lastly, we calculated the friction tensor, , of the different tracers in the first linear regime at small forces. To determine the diagonal elements, which correspond to the friction experienced by the tracer in the directions parallel and perpendicular to **n**, we analyzed the effect of *F*_∥_ and *F*_⊥_ on the tracer particle (see frames (a) of Figures 2 and 6). On the other hand, the nondiagonal elements represent the coupling between γ_eff,∥_ and γ_eff,⊥_ in the diffusion of the probe. We decomposed the tracer velocities caused by the force *F*_45°_ exerted on it and found that the nondiagonal components of  are negligible, with values less than 15.7% of the other terms (see Table S2 in the Supporting Information).

**Figure 7:**
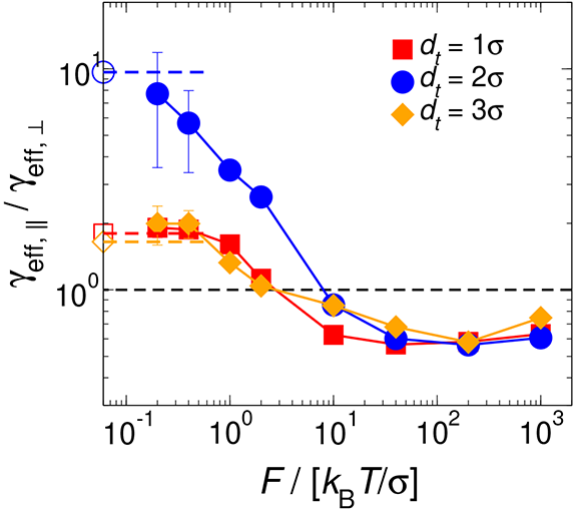
Effective friction coefficient ratio *R*_*d*_*t*__ = γ_eff,∥_/γ_eff,⊥_ of hard rods in the Sm phase in the low- and high-force regimes as a function of the tracer diameter and the external force, parallel and perpendicular to the nematic director **n**. Empty symbols indicate the results obtained from passive MR (*F* = 0) from ref (45). Solid and dashed lines are guides for the eye.

In Figure 7, we present the ratio *R*_*d*_*t*__ = γ_eff,∥_/γ_eff,⊥_ between the friction coefficients parallel and perpendicular to **n** as probed by tracers of different sizes at different intensities of the external forces *F*_∥_ and *F*_⊥_. In the case of low to intermediate forces, the tracer with a diameter of 2σ exhibits a higher *R*_*d*_*t*__ compared to the other two sizes. This phenomenon can be attributed to the tracer’s tendency to predominantly occupy the interlayered spaces, as clearly demonstrated by the density profiles of rods illustrated in Figure 3 and Figure S3 of the Supporting Information. Such a preference leads to a higher frequency of collisions between the tracer and rods, especially causing a notable accumulation of bath particles around the probe when it is dragged by *F*_∥_, in contrast to *F*_⊥_. The accumulation of rods opposes the diffusion of the tracer through the layered arrangement of particles, thereby increasing its perceived friction coefficient, γ_eff,∥_. Subsequently, when *d*_*t*_ = 1σ and *d*_*t*_ = 3σ, the tracer detects a lower density of layered rods at its front (see Figure 3). This allows for less hindrance to its diffusion than in the case of *d*_*t*_ = 2σ, resulting in a decrease in *R*_*d*_*t*__. Interestingly, tracers with sizes 1σ and 3σ exhibit a uniform behavior (*R*_*d*_*t*__ ≃ 2) at forces *F* < 10 *k*_B_*T*/σ. However, it should be noted that for intermediate tracer sizes (*d*_*t*_ = 1.5σ and *d*_*t*_ = 2.5σ) in the context of passive MR, the values of the ratio result in *R*_*d*_*t*_=1.5σ_ ≈ 7.5 and *R*_*d*_*t*_=2.5σ_ ≈ 4.8, respectively.^45^ Therefore, based on the present results, we cannot claim that the similarity in friction ratios for *d*_*t*_ = 1σ and *d*_*t*_ = 3σ stems from a physical cause or is merely a chance occurrence in the numerical data.

Finally, following a decrease at intermediate forces, it is observed that at large forces *R*_*d*_*t*__ reaches a constant value for all tracer sizes. Furthermore, regardless of the tracer diameter *d*_*t*_, it is found that *R*_*d*_*t*__ < 1. The presence of the tracer activates a diffusion channel in the adjacent layers,^31^ which, together with *F*_∥_ pulling the probe, facilitates its movement through the layers and even causes a loss of perception of the microstructured surrounding fluid, as shown in Figure 3 (bottom row). Moreover, an increase in the number of collisions between the tracer and the surrounding particles is anticipated as the angle between the applied force and **n** rises. Specifically, for *F*_∥_, the thin regions of rods around the probe are less prominent than for *F*_⊥_ (see Figure 3 and Figure S3 of the Supporting Information), allowing for a smoother motion of the tracer particle along **n** and resulting in γ_eff,∥_ < γ_eff,⊥_. It should be noted that the tracer’s low perception of the microstructured fluid, when the external force is sufficiently large, produces values of γ_eff,∥_ and γ_eff,⊥_ that, when directly compared, eliminate the tracer size effect and reveal a single curve for *R*_*d*_*t*__, indicating a universal behavior of the active MR in Sm fluids in large-force regimes.

## 4 Conclusions

This work investigates the local rheological properties of structured fluids in liquid-crystalline phases by using fixed-forced active microrheology. Specifically, we focus on the linear and nonlinear viscoelastic responses of smectic liquid crystals of colloidal hard rods exhibiting orientational and positional order. By employing DMC simulations, we explored the influence of the tracer size and direction of the external force on the effective friction coefficient γ_eff_ of the host system. In general, while γ_eff_ behaves nonlinearly at intermediate forces, a linear response of γ_eff_ is observed at low and high forces. More specifically, when the force *F*_∥_ is low and aligned to the nematic director **n**, the fluid’s viscoelastic behavior remains linear due to the dominance of thermal motion over advection. As a result, the effective viscosity γ_eff,∥_ remains constant. While this linear response is observed for tracers of sizes *d*_*t*_ = 1σ and 3σ, the intense confinement in between the Sm layers for the tracer with *d*_*t*_ = 2σ hampers it from diffusing throughout the system uniformly and probing the microrheological properties of the bath. At intermediate forces, advection is comparable to diffusion, and a force-thinning regime emerges, which reveals itself in a nonlinear response of γ_eff,∥_ that decreases with *F*_∥_. Finally, at large forces advection of bath particles dominates its Brownian response, and a second plateau γ_eff,∥_ is observed. Each of these behaviors has been reported in theoretical,^17−19,22,59^ simulation,^12,18,21,23,25,26^ and experimental^57,58^ studies on colloidal suspensions.

When the external force is oriented diagonal (*F*_45°_) or perpendicular (*F*_⊥_) to **n**, a qualitatively similar behavior of the effective friction to the parallel case is observed: a Newtonian plateau at small and large forces and a nonlinear regime at intermediate forces. Additionally, we have also examined the behavior of the effective friction by decomposing *F*_45°_ into its components parallel (*F*_45°,∥_) and perpendicular (*F*_45°,⊥_) to **n**. In the parallel case, the values of γ_eff,∥_ as obtained from applying *F*_∥_ to the tracer are recovered. On the other hand, from the analysis of *F*_45°,⊥_ we observe that γ_eff,⊥_ closely agrees with that calculated from *F*_⊥_. While the agreement is excellent within a low-force regime, for intermediate and large forces the differences are systematically larger. In the absence of external forces, the tracers are more likely to stay and diffuse in the interstitial planes between the layers.^31,45,48,61,62^ Upon applying an external force *F*_⊥_ on the tracers, the particles are driven to explore the interlayer regions more intensively. By contrast, the action of *F*_45°_ favors a penetration of the tracer in the Sm layers and a more effective probing of the local effective friction. Nevertheless, the differences found for the case *d*_*t*_ = 3σ are evidently marginal under the two analyses given the intrinsic ability of the tracer to travel throughout the layered rods as pointed out in our former work.^45^ In light of these results, the presence of an external force that is neither entirely parallel or perpendicular to **n** provides an advantage for the tracer to overcome the energetic barriers imposed by correlations between the tracer size and the positional or orientational orders dictated by the particles of the Sm fluid. In this way, the tracer is capable of probing more extensively the local viscoelastic properties in the linear and nonlinear regimes of the host fluid irrespective of the tracer and system characteristic lengths.

We have also analyzed the ratio, *R*_*d*_*t*__, between the friction coefficients γ_eff,∥_ and γ_eff,⊥_ for a number of external forces *F*_∥_ and *F*_⊥_, respectively. At low forces, we observe a dominance of γ_eff,∥_ over γ_eff,⊥_ (*R*_*d*_*t*__ > 1) as a consequence of the tracer being hindered to penetrate the Sm layers and diffuse along **n**. In particular, we observe a significant increase in the value of *R*_*d*_*t*__ for *d*_*t*_ = 2σ. This increase in *R*_*d*_*t*__ is caused by the strong preference of the tracer to occupy the quasi-2D regions between the layers.^45^ This behavior reduces the frequency of successful attempts to penetrate and diffuse through the layered rods, thereby resulting in a marked increase in γ_eff,∥_ compared to γ_eff,⊥_. Conversely, we found a less marked increase in *R*_*d*_*t*__ at *d*_*t*_ = 1σ and *d*_*t*_ = 3σ. In the former case, the activation of diffusion channels favors the tracer’s movement parallel to **n**,^31^ whereas in the latter, the probe deforms the rods of the adjacent layers, making it easier for the tracer to enter and diffuse within the Sm layers. Both scenarios lead to a γ_eff,∥_ that is still higher than γ_eff,⊥_ but significantly lower than γ_eff,∥_ for *d*_*t*_ = 2σ. Finally, at moderate and large forces, the value of *R*_*d*_*t*__ decreases until it reaches a constant value of *R*_*d*_*t*__ ≃ 0.6. At high *F*_∥_, the tracer’s probing of the microstructural order of the host fluid is reduced, and γ_eff,∥_ scales proportionally with *d*_*t*_. Similarly, for *F*_⊥_, an increase in the number of rods in contact with the probe occurs with an increase in *d*_*t*_, which results in a size-dependent increase in γ_eff,⊥_. In this case, γ_eff,⊥_ exceeds γ_eff,∥_ for all tracer sizes. Linking these resulting effective frictions yields a single *R*_*d*_*t*__ response curve that is independent of the tracer size and marks a universal behavior of the active MR at large forces for Sm-phase LC systems.

## Supporting Information Available

DMC simulation details for the different external forces and tracer sizes; orientation correlation functions for rods at different values of external force **F**_∥_ and tracer particle diameter *d*_*t*_; density distribution of rod particles around the tracers for external forces **F**_⊥_ and **F**_45°_; and effective friction coefficients parallel and perpendicular to the nematic director and their correlations at low forces (PDF)
