# Supplementary material for: Insight into the Viscoelasticity of Self-Assembling Smectic Liquid Crystals of Colloidal Rods from Active Microrheology Simulations
Source: J Chem Theory Comput. 2023 Jun 30;20(4):1579–89. doi: 10.1021/acs.jctc.3c00356 (PMC10902840; doi:10.1021/acs.jctc.3c00356)
Supplement: Supplementary file 1 — ct3c00356_si_001.pdf [file ct3c00356_si_001.pdf]

Supporting Information

An Insight into the Viscoelasticity of Self-Assembling Smectic Liquid Crystals of Colloidal Rods from Active Microrheology Simulations

Fabián A. García Daza*[,1,2], Antonio M. Puertas[3], Alejandro Cuetos[1], and Alessandro Patti†[,4,2]

[1]Department of Physical, Chemical and Natural Systems, Pablo de Olavide University, 41013, Sevilla, Spain
[2]Department of Chemical Engineering, The University of Manchester, Manchester M13 9PL, UK
[3]Department of Chemistry and Physics, University of Almería, 04120, Almería, Spain
[4]Department of Applied Physics, University of Granada, Fuente Nueva s/n, 18071 Granada, Spain

*fagardaz@upo.es
†apatti@ugr.es

S1. Details of the Spherical Tracers and Hard Rods Systems

In this work, the systems studied consisted of one spherical particle with diameter d_t embedded in a bath of $N_r = 1400$ rod-like particles in smectic (Sm) phase with length-to-diameter ratio $L^* \equiv L/\sigma = 5$. In Table S1, we report the external force intensity and direction with respect to the nematic director $\mathbf{n}$, the tracer size d_t, elementary time steps $\delta t_{\mathrm{MC},r}$ and $\delta t_{\mathrm{MC},t}$ in units of τ, and acceptance rates $\mathcal{A}_r$ and $\mathcal{A}_t$ of rods and tracer particles, respectively.

Table S1: Details of the systems studied in this work. The external force intensity, diameter d_t, MC time steps $\delta t_{\mathrm{MC},r}$ and $\delta t_{\mathrm{MC},t}$ of the rods and tracer particles are presented with their acceptance rates $\mathcal{A}_r$ and $\mathcal{A}_t$, respectively.

$\mathbf{F}_{\parallel}$: Forces parallel to $\mathbf{n}$					
$F_{\parallel}/(k_{\mathrm{B}}T\sigma^{-1})$	d_t/σ	$\delta t_{\mathrm{MC},r}/\tau$	$\delta t_{\mathrm{MC},t}/\tau$	$\mathcal{A}_r$	$\mathcal{A}_t$
0.2	1	$5 \cdot 10^{-3}$	$4.67 \cdot 10^{-3}$	0.748	0.870
0.4	1	$5 \cdot 10^{-3}$	$4.68 \cdot 10^{-3}$	0.748	0.869
1	1	$5 \cdot 10^{-3}$	$4.76 \cdot 10^{-3}$	0.748	0.858
2	1	$5 \cdot 10^{-3}$	$4.92 \cdot 10^{-3}$	0.747	0.845
10	1	$1 \cdot 10^{-3}$	$1.11 \cdot 10^{-3}$	0.877	0.861
40	1	$1 \cdot 10^{-4}$	$1.18 \cdot 10^{-4}$	0.959	0.881
200	1	$2 \cdot 10^{-6}$	$2.21 \cdot 10^{-6}$	0.994	0.935
1000	1	$1 \cdot 10^{-7}$	$1.10 \cdot 10^{-7}$	0.998	0.935
0.2	2	$1 \cdot 10^{-3}$	$1.05 \cdot 10^{-3}$	0.878	0.892
0.4	2	$1 \cdot 10^{-3}$	$1.05 \cdot 10^{-3}$	0.878	0.896
1	2	$1 \cdot 10^{-3}$	$1.06 \cdot 10^{-3}$	0.878	0.888
2	2	$1 \cdot 10^{-3}$	$1.06 \cdot 10^{-3}$	0.877	0.890
10	2	$2 \cdot 10^{-3}$	$2.46 \cdot 10^{-3}$	0.831	0.782
40	2	$2 \cdot 10^{-4}$	$2.45 \cdot 10^{-4}$	0.943	0.845
200	2	$4 \cdot 10^{-6}$	$4.50 \cdot 10^{-6}$	0.992	0.921
1000	2	$2 \cdot 10^{-7}$	$2.22 \cdot 10^{-7}$	0.998	0.929
0.2	3	$1 \cdot 10^{-3}$	$1.16 \cdot 10^{-3}$	0.877	0.836
0.4	3	$1 \cdot 10^{-3}$	$1.16 \cdot 10^{-3}$	0.876	0.840
1	3	$1 \cdot 10^{-3}$	$1.17 \cdot 10^{-3}$	0.877	0.841
2	3	$1 \cdot 10^{-3}$	$1.17 \cdot 10^{-3}$	0.877	0.842

10	3	$3 \cdot 10^{-3}$	$4.92 \cdot 10^{-3}$	0.798	0.661
40	3	$3 \cdot 10^{-4}$	$3.79 \cdot 10^{-4}$	0.93	0.821
200	3	$6 \cdot 10^{-6}$	$6.73 \cdot 10^{-6}$	0.99	0.913
1000	3	$3 \cdot 10^{-7}$	$3.36 \cdot 10^{-7}$	0.998	0.925
$\mathbf{F}_\perp$: Forces perpendicular to $\mathbf{n}$					
$F_\perp/(k_\mathrm{B}T\sigma^{-1})$	d_t/σ	$\delta t_{\mathrm{MC},r}/\tau$	$\delta t_{\mathrm{MC},t}/\tau$	$\mathcal{A}_r$	$\mathcal{A}_t$
0.2	1	$5 \cdot 10^{-3}$	$4.63 \cdot 10^{-3}$	0.747	0.872
0.4	1	$5 \cdot 10^{-3}$	$4.66 \cdot 10^{-3}$	0.747	0.870
1	1	$5 \cdot 10^{-3}$	$4.70 \cdot 10^{-3}$	0.748	0.865
2	1	$5 \cdot 10^{-3}$	$4.80 \cdot 10^{-3}$	0.747	0.861
10	1	$1 \cdot 10^{-3}$	$1.07 \cdot 10^{-3}$	0.877	0.879
40	1	$1 \cdot 10^{-4}$	$1.14 \cdot 10^{-4}$	0.959	0.897
200	1	$2 \cdot 10^{-6}$	$2.20 \cdot 10^{-6}$	0.994	0.938
1000	1	$1 \cdot 10^{-7}$	$1.10 \cdot 10^{-7}$	0.999	0.938
0.2	2	$1 \cdot 10^{-3}$	$1.05 \cdot 10^{-3}$	0.878	0.890
0.4	2	$1 \cdot 10^{-3}$	$1.05 \cdot 10^{-3}$	0.878	0.890
1	2	$1 \cdot 10^{-3}$	$1.06 \cdot 10^{-3}$	0.878	0.889
2	2	$1 \cdot 10^{-3}$	$1.06 \cdot 10^{-3}$	0.878	0.885
10	2	$2 \cdot 10^{-3}$	$2.41 \cdot 10^{-3}$	0.831	0.793
40	2	$2 \cdot 10^{-4}$	$2.38 \cdot 10^{-4}$	0.943	0.868
200	2	$4 \cdot 10^{-6}$	$4.44 \cdot 10^{-6}$	0.992	0.928
1000	2	$2 \cdot 10^{-7}$	$2.22 \cdot 10^{-7}$	0.998	0.930
0.2	3	$1 \cdot 10^{-3}$	$1.16 \cdot 10^{-3}$	0.877	0.842
0.4	3	$1 \cdot 10^{-3}$	$1.16 \cdot 10^{-3}$	0.877	0.842
1	3	$1 \cdot 10^{-3}$	$1.15 \cdot 10^{-3}$	0.877	0.841
2	3	$1 \cdot 10^{-3}$	$1.17 \cdot 10^{-3}$	0.878	0.834
10	3	$3 \cdot 10^{-3}$	$4.95 \cdot 10^{-3}$	0.797	0.673
40	3	$3 \cdot 10^{-4}$	$3.73 \cdot 10^{-4}$	0.930	0.841
200	3	$6 \cdot 10^{-6}$	$6.69 \cdot 10^{-6}$	0.988	0.922
1000	3	$3 \cdot 10^{-7}$	$3.34 \cdot 10^{-7}$	0.998	0.929
$\mathbf{F}_{45^\circ}$: Forces oriented 45° with respect $\mathbf{n}$					
$F_{45^\circ}/(k_\mathrm{B}T\sigma^{-1})$	d_t/σ	$\delta t_{\mathrm{MC},r}/\tau$	$\delta t_{\mathrm{MC},t}/\tau$	$\mathcal{A}_r$	$\mathcal{A}_t$
0.2	1	$5 \cdot 10^{-3}$	$4.63 \cdot 10^{-3}$	0.748	0.875
0.4	1	$5 \cdot 10^{-3}$	$4.66 \cdot 10^{-3}$	0.748	0.872

1	1	$5 \cdot 10^{-3}$	$4.72 \cdot 10^{-3}$	0.748	0.862
2	1	$5 \cdot 10^{-3}$	$4.92 \cdot 10^{-3}$	0.748	0.845
10	1	$1 \cdot 10^{-3}$	$1.12 \cdot 10^{-3}$	0.877	0.867
40	1	$1 \cdot 10^{-4}$	$1.17 \cdot 10^{-4}$	0.959	0.884
200	1	$2 \cdot 10^{-6}$	$2.21 \cdot 10^{-6}$	0.994	0.936
1000	1	$1 \cdot 10^{-7}$	$1.10 \cdot 10^{-7}$	0.999	0.933
0.2	2	$1 \cdot 10^{-3}$	$1.05 \cdot 10^{-3}$	0.877	0.890
0.4	2	$1 \cdot 10^{-3}$	$1.05 \cdot 10^{-3}$	0.878	0.893
1	2	$1 \cdot 10^{-3}$	$1.05 \cdot 10^{-3}$	0.878	0.891
2	2	$1 \cdot 10^{-3}$	$1.06 \cdot 10^{-3}$	0.878	0.888
10	2	$2 \cdot 10^{-3}$	$2.45 \cdot 10^{-3}$	0.831	0.792
40	2	$2 \cdot 10^{-4}$	$2.42 \cdot 10^{-4}$	0.943	0.858
200	2	$4 \cdot 10^{-6}$	$4.47 \cdot 10^{-6}$	0.992	0.927
1000	2	$2 \cdot 10^{-7}$	$2.22 \cdot 10^{-7}$	0.998	0.927
0.2	3	$1 \cdot 10^{-3}$	$1.14 \cdot 10^{-3}$	0.877	0.839
0.4	3	$1 \cdot 10^{-3}$	$1.14 \cdot 10^{-3}$	0.878	0.846
1	3	$1 \cdot 10^{-3}$	$1.15 \cdot 10^{-3}$	0.876	0.839
2	3	$1 \cdot 10^{-3}$	$1.17 \cdot 10^{-3}$	0.878	0.831
10	3	$3 \cdot 10^{-3}$	$4.93 \cdot 10^{-3}$	0.798	0.662
40	3	$3 \cdot 10^{-4}$	$3.77 \cdot 10^{-4}$	0.930	0.829
200	3	$6 \cdot 10^{-6}$	$6.73 \cdot 10^{-6}$	0.990	0.919
1000	3	$3 \cdot 10^{-7}$	$3.33 \cdot 10^{-7}$	0.998	0.924

S2. Orientation Correlation Functions of Hard-Rods in Smectic Phase

Figure S1 depicts the orientational correlation functions $E_2(v)$ for bath particles in volumes v at different values of the force parallel ($F_{\parallel}$) to the nematic director ($\mathbf{n}$) and tracer diameter d_t. The changes in $E_2(v)$ for different values of $F_{\parallel}$ are estimated from:

$$E_2(v) = \left\langle \frac{1}{N_r(v,t)} \sum_{i=1}^{N_r(v,t)} \frac{1}{2} \left[3 \left(\hat{\mathbf{u}}_i(v,t) \cdot \mathbf{n} \right)^2 - 1 \right] \right\rangle , \tag{S1}$$

where $\hat{\mathbf{u}}_i(v,t)$ refers to the unit orientation vectors of $N_r(v,t)$ rods in volume v at time t. If the rods are parallel to $\mathbf{n}$, then $E_2(v) \approx 1$. On the other hand, if the rods orientations are perpendicular to the nematic director then $E_2(v) \approx -1/2$.

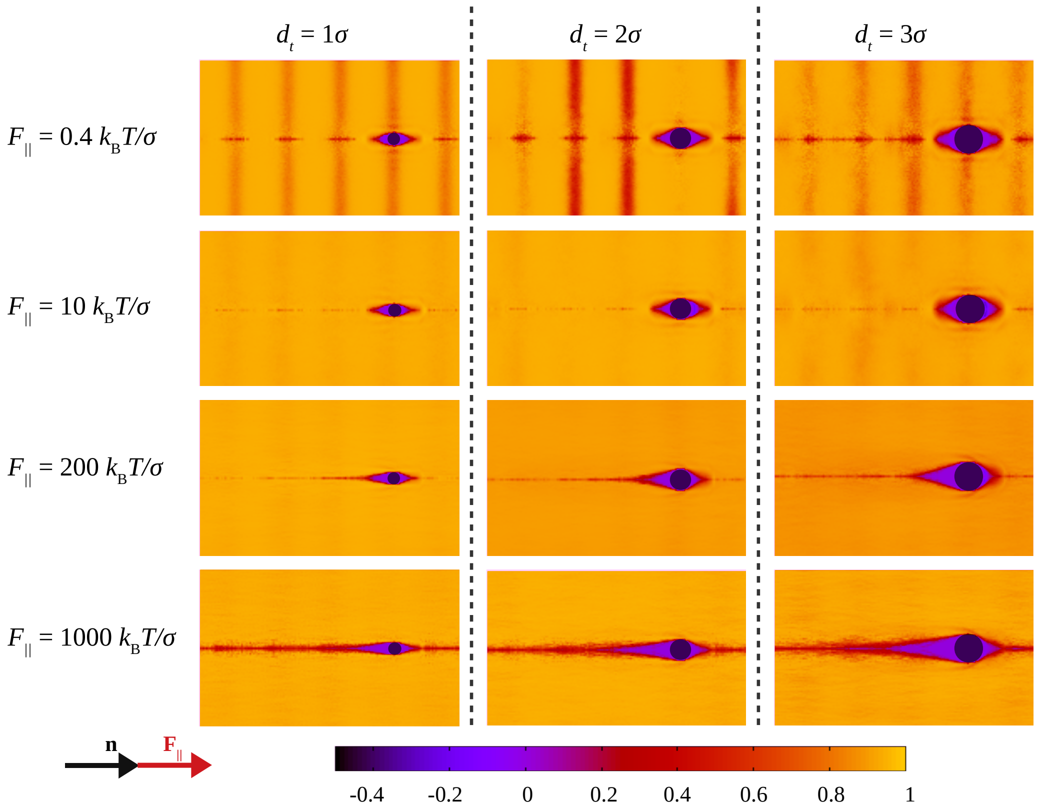

Figure S1: (colour on-line) Orientational correlation functions of a system of hard rods in Sm phase at $\phi = 0.51$ around a tracer with diameter 1σ (left column), 2σ (center column), and 3σ (right column). The tracer particle is pulled by an external force $\mathbf{F}_{||}$ parallel to the nematic director $\mathbf{n}$. The color palette is shown at the bottom of the figure.

S3. Trajectories of a Spherical Tracer in a Bath of Rods in Smectic Phase

Bath density distribution for a system of hard rods and a tracer particle are shown in Figures S2 and S3 for external forces oriented 45° and 90° with respect to the nematic director **n**, respectively. While rods are monodisperse and have length-to-diameter ratio $L^* = 5$, the tracers are modeled as hard spheres with diameters between 1σ and 3σ.

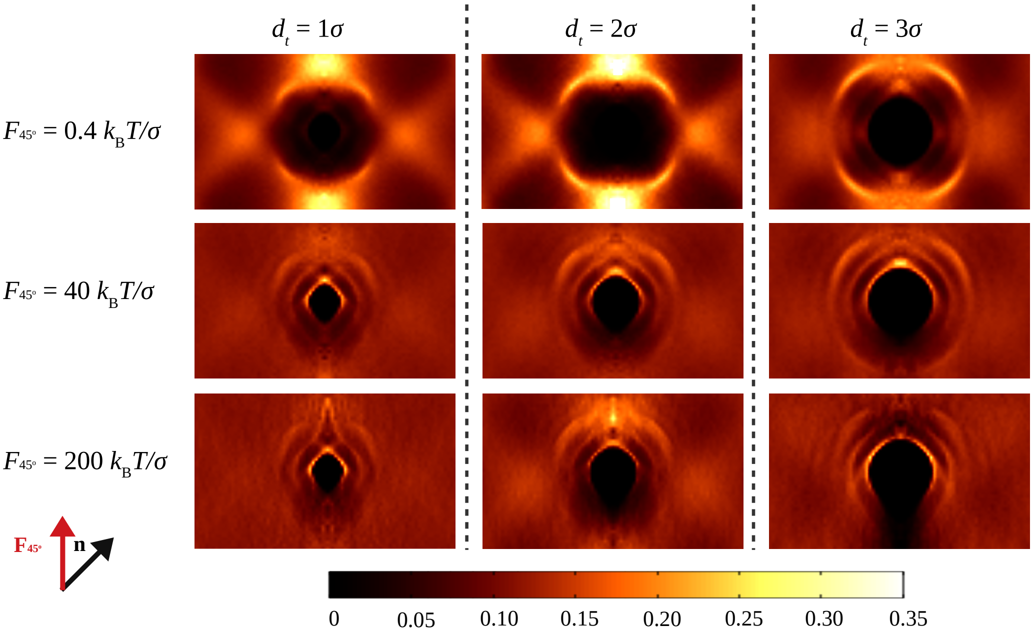

Figure S2: (colour on-line) Density maps of a system of hard rods in Sm phase at $\phi = 0.51$ around a tracer with diameter 1σ (left column), 2σ (center column), and 3σ (right column). The tracer particle is pulled by an external force $\mathbf{F}_{45^\circ}$ oriented 45° with respect to the nematic director **n**. The color palette is shown at the bottom of the figure.

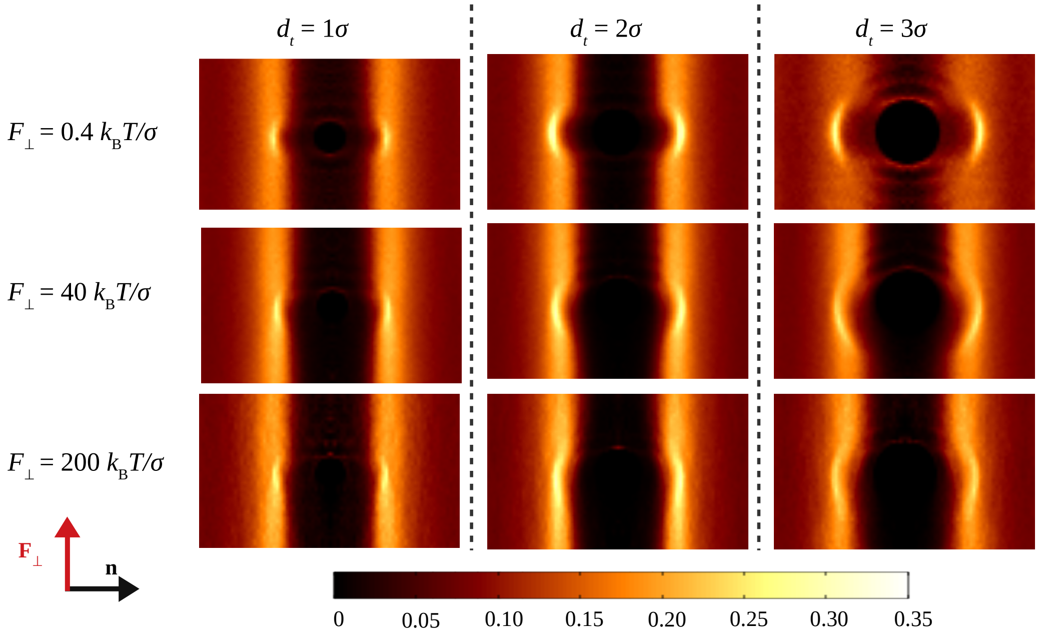

Figure S3: (colour on-line) Density maps of a system of hard rods in Sm phase at $\phi = 0.51$ around a tracer with diameter 1σ (left column), 2σ (center column), and 3σ (right column). The tracer particle is pulled by an external force $\mathbf{F}_\perp$ perpendicular to the nematic director $\mathbf{n}$. The color palette is shown at the bottom of the figure.

S4. Friction Tensor in Smectic Phases of Rod-Shaped Particles Probed by a Spherical Tracer

The effective friction tensor of a bath of rods probed by a spherical tracer reads:

$$\vec{\vec{\gamma}}_{\text{eff}} = \begin{bmatrix} \gamma_{\text{eff},\parallel} & \gamma_{\text{eff},\parallel\perp} \\ \gamma_{\text{eff},\perp\parallel} & \gamma_{\text{eff},\perp} \end{bmatrix} \tag{S2}$$

where the elements $\gamma_{\text{eff},\parallel}$ and $\gamma_{\text{eff},\perp}$ represent the frictions experienced by the tracer along and transverse to the nematic director, repectively. The non-diagonal elements, $\gamma_{\text{eff},\parallel\perp}$ and $\gamma_{\text{eff},\perp\parallel}$, refer to the correlations between the effective frictions in the principal directions of motion. While $\gamma_{\text{eff},\parallel}$ and $\gamma_{\text{eff},\perp}$ are calculated from the analysis of the effect of $\mathbf{F}_\parallel$ and $\mathbf{F}_\perp$ at low intensities on the tracer particle, the non-diagonal elements of $\vec{\vec{\gamma}}_{\text{eff}}$ are derived by decomposing the parallel and perpendicular contributions of $\mathbf{F}_{45^\circ}$ on the tracer. Table S2 shows the diagonal and non-diagonal effective frictions of a bath of hard rods in the Sm phase as probed by tracer particles of different sizes at low forces.

Table S2: List of the effective frictions perceived by a tracer particle with diameter d_t. The units of the friction coefficients are given by $\gamma_0 = 3\pi\eta_s d_t$, and η_s is the viscosity of the medium.

d_t/σ	$\gamma_{\text{eff},\parallel}/\gamma_0$	$\gamma_{\text{eff},\perp}/\gamma_0$	$\gamma_{\text{eff},\parallel\perp}/\gamma_0$	$\gamma_{\text{eff},\perp\parallel}/\gamma_0$
1	8.1(0.4)	5.25(0.02)	0.27(0.01)	-0.5(0.3)
2	68(23)	9.5(0.2)	1.3(0.3)	-1.3(0.1)
3	38(3)	19(2)	-2.3(0.5)	-3(1)
